# Energy: Protein Ratio in Ruminants: Insights from the Intragastric Infusion Technique

**DOI:** 10.3390/ani11092700

**Published:** 2021-09-15

**Authors:** Denis J. Meehan, Ana R. J. Cabrita, Margarida R. G. Maia, António J. M. Fonseca

**Affiliations:** 1 REQUIMTE, LAQV, ICBAS, Instituto de Ciências Biomédicas de Abel Salazar, Universidade do Porto, Rua Jorge Viterbo Ferreira, 4050-313 Porto, Portugal; djmeehan@icbas.up.pt (D.J.M.); arcabrita@icbas.up.pt (A.R.J.C.); mrmaia@icbas.up.pt (M.R.G.M.); 2 UTAD, Universidade de Trás-os-Montes e Alto Douro, Quinta de Prados, 5000-801 Vila Real, Portugal

**Keywords:** energy:protein, intragastric infusion technique, nitrogen, volatile fatty acids

## Abstract

**Simple Summary:**

One key question that has confounded nutritional scientists for years is how the ruminant responds metabolically with respect to energy and nitrogen utilisation when no exogenous energy is consumed. Fasting metabolism studies using the intragastric infusion technique (IIT) showed this to be a glucose-deficient state characterised by elevated nitrogen excretion and heat production. However, modern feeding systems continue to adopt fasting as the basis for measuring utilisation efficiency of nutritionally balanced diets, giving rise to the false concept of greater feed utilisation below than above energy maintenance. Another IIT finding was that given the animal’s genetic capacity for protein accretion and provided a rumen undegradable protein is fed, ruminants do not catabolise amino acids as an energy source but instead retain these to attain substantial gains in tissue protein deposition, fuelled by endogenous energy reserves. This suggests that endogenous fat reserves could be used to retain body protein when feed supplies are scarce or of poor nutritive value and questions the need to use high-energy diets in the finishing pre-slaughter period. Moreover, body protein and body fat deposition were also shown to be negatively correlated, contradicting current feeding systems which assume that nitrogen retention is always negative in an underfeeding situation.

**Abstract:**

Studies on energy:protein ratio in ruminants are constrained by rumen fermentation since it governs nutrient metabolism and the ratio of energy:protein yielding nutrients available for absorption. By circumventing rumen fermentation, the total intragastric infusion technique (IIT) allowed objective quantification of maintenance energy and protein requirements, volatile fatty acid utilisation efficiency, efficiency of energy utilisation for maintenance (K_m_) and growth (K_f_) and the origin of N retention responses to independent variation of energy and protein intake. This review outlines the key IIT findings and whether they are reflected in current feeding systems with implications for different production systems worldwide. Maintenance energy requirements are similar to those derived from comparative slaughter but maintenance N requirements are significantly lower. No differences in utilisation efficiency exist between acetic, propionic and butyric acids. At low energy intakes, endogenous energy reserves are utilised to retain amino acids and fuel substantial tissue protein gains. The use of fasting metabolism to measure the utilisation of nutritionally balanced diets is questioned since it is a glucose-deficient state. Inter-species differences in glucose metabolism appear to exist, suggesting that glucose requirements may be higher in cattle than sheep. The difficulty in predicting nutrient requirements, particularly protein, with any one technique is highlighted.

## 1. Introduction

Energy:protein (E:P) ratio in ruminants is complex due to the myriad of interactions at ruminal and post-ruminal absorption and metabolism levels which influence the response to modulation of dietary energy and protein supply. This has presented many challenges to nutritionists in interpretation of experimental findings related to energy metabolism, including absorption, metabolism and utilisation of volatile fatty acids (VFA), and animal responses to manipulations of dietary energy and protein supply.

The fermentation process of structural and non-structural carbohydrates to VFA by microbial action in the rumen involves an interdependency of fermentable energy and microbial protein production [1]. This has a number of implications for the ruminant. Energy retention efficiency is not constant between carbohydrate sources and appears negatively correlated to the molar proportion of acetic acid produced in the rumen, derived from high-fibre diets. Due to the obligatory requirement for nitrogen (N) by rumen microbes in rumen fermentation [2,3], the ratio of E:P nutrients available for absorption becomes relatively constant, irrespective of intake [4] since the amino acids (AA) absorbed in the intestine are proportional to the energy fermented in the rumen [5]. However, the E:P ratio absorbed can be reduced in diets containing ingredients with slower rates of ruminal degradation where proteins from high concentrate [6] or high roughage diets [7,8] flow intact from the rumen. Similarly, diets which support high protozoa numbers reduce the ratio of E:P absorbed by ruminants [9], presumably through predation of rumen bacteria by protozoa reducing microbial mass flowing from the rumen [10]. Additionally, the quantity and profile of nutrients ultimately absorbed at the small intestine bears little resemblance to that ingested as dietary protein [11,12]. At low (sub-maintenance and maintenance) levels of feeding little dietary protein evades rumen degradation due to prolonged rumen retention times [6]. Conversely, high feeding levels increase rumen outflow rate [4], reduce protein degradation in the rumen [2] and increase N flow to the intestine [4] to increase protein retention. This suggests that the observed increase in protein/N retention in response to an increased energy intake [13,14] reflects a response to an increased AA supply at the small intestine rather than an energy effect per se [11]. Furthermore, the deposition of body protein and fat are usually positively correlated in fed animals [15]. Estimating maintenance N requirements in normally fed ruminants involves the use of N-free diets but in these situations microbial N requirements are not met, rumen organic matter fermentation falls and consequently feed intake grinds to a halt [2].

The first comprehensive model to describe the relationship between energy and protein intake to protein retention in ruminants and non-ruminants was provided by Balch [13]. This model presumes a series of linear–curvilinear responses relating protein and energy intake to N retention, and assumes that N retention at a given N intake depends on the level of energy intake, provided N intake is in excess of animal requirement, thus indicating that ruminant E:P requirements cannot be determined by simply altering the energy and protein content of diets. Results for protein retained in the animal body will vary depending on how the specific dietary ingredients alter the flow of dietary and microbial protein from the rumen and energy absorption through the rumen wall and digestive tract [2].

The development of the total intragastric infusion technique (IIT) allowed ruminant tissue requirements for energy and protein to be determined through by-passing rumen fermentation with entire diets [16] since this technique permitted true responses in protein retention (and tissue AA requirements) to be quantified as a function of energy and protein intake. Indeed, the IIT was the first technique to permit definitive work to be undertaken on the cornerstones of ruminant nutrition, namely VFA utilisation efficiency, the efficiency of energy utilisation for maintenance (K_m_) and growth (K_f_), and tissue protein requirements. Furthermore, by allowing independent variation of the E:P ratio of single-nutrient infusions as either abomasal casein, ruminal VFA or entire diets at sub-maintenance, maintenance and supra-maintenance energy intake, the animals’ metabolic response in N retention with respect to energy and glucogenic precursor supply per se could be evaluated.

The objective of this review is to revisit the key findings from the IIT with respect to E:P metabolism and to re-evaluate some concepts assumed in current feeding system with implications for different production systems worldwide.

## 2. The Total Intragastric Infusion Technique

**Figure 1:**
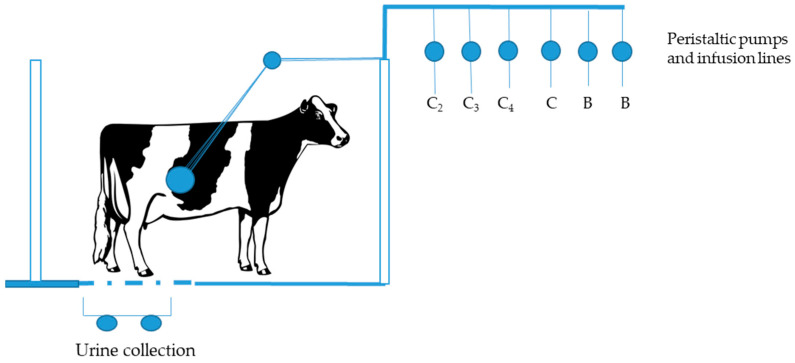
Schematic representation of the total intragastric infusion technique. C_2_—acetic acid; C_3_—propionic acid; C_4_—butyric acid; C—casein; B—buffer.

Energy utilisation in animals with a functioning rumen fermentation, through feeding basal diets supplemented with VFA acids infusion or VFA salts, were extremely difficult to assess due to the impossibility of controlling the VFA composition absorbed, interference with digestion and metabolism of the basal diet and inappetence [17]. Development of the total IIT made definitive work on energy utilisation, VFA utilisation and tissue protein requirements possible. A diagrammatic representation of the system is shown in Figure 1.

For the first time, sheep [18] and cattle [16] could be entirely maintained on ruminal VFA and abomasal protein and these nutrients could be infused independently and at will [19] for longer periods of up to three months [18]. Central to this technique was the use of a rumen buffer which rectified the problems of electrolyte imbalance [20], pH [18] and osmotic pressure [16].

Since animals could be maintained on N-free infusates, the IIT also leveraged the knowledge on urinary purine derivative excretion [21] and endogenous/basal N excretion [2,22]. In addition, this technique yielded insights into urinary creatinine excretion [23], rumen osmotic pressure [24], water kinetics [25], rumen VFA absorption [26] and utilisation efficiency of rumen microbial N [27]. Although a regressed and thin-walled intestine was noticed, rumen papillae length and mitotic index were unchanged [18,19]. No problems were documented relating to ruminant species or physiological state [16]. Lactating dairy cow reproduction was generally normal, with normal oestrus cycle, service-to-conception interval and fully healthy calves being born on successive gestations [16] and with lambs, carcass fat cover, composition and colour were similar to that of normally fed lambs [18]. The main findings from the IIT with respect to E:P metabolism will be reviewed as follows.

### 2.1. Maintenance Requirements for Energy and Protein

Maintenance energy requirements for ruminants were estimated using the IIT at 450 kJ/kg metabolic bodyweight (W^.75^) which is broadly similar to those derived from the comparative slaughter technique (Table 1).

Maintenance N requirements are based on quantifying endogenous N losses which are composed of endogenous urinary N (EUN) and metabolic faecal N excretion. Determined at maintenance energy and zero N infused, EUN excretion quantifies the net minimum N requirement for maintenance [30,31] since metabolic faecal N excretion is virtually absent in IIT [32] (Table 2).

Maintenance N requirements determined by IIT are often lower than those estimated by normally fed animals although similar to the values reported in milk-fed lambs using comparative slaughter [34]. These values contrast with ARC [1] where EUN values are 3–4 times greater. This discrepancy has been attributed to the absence of endogenous N recycling to the rumen on infusion and the negligible production of metabolic faecal N. Metabolic faecal N and EUN production are negatively correlated [2,22], the former increasing with increased hind-gut fermentation [30,35]. Endogenous protein can account for up to 16% of total flow at the small intestine [36] and endogenous N secretions can constitute 30% of total digestive tract protein synthesis in dairy cows [37].

### 2.2. Volatile Fatty Acid Utilisation as a Function of Energy Level (K_f_) and Individual VFA Composition

The efficiency of utilisation of retained energy (K_f_) obtained from IIT is shown in Table 3. These data were obtained using indirect open-circuit calorimetry to calculate heat production at VFA energy infusions ranging from maintenance to twice maintenance requirements.

Compared to feeding trial results (Table 4), it can be seen that with the exception of data from Preston et al. [41], the values are generally in broad agreement with those from the IIT.

No pronounced variations were observed with K_f_ obtained from ITT standing in the range of 0.55–0.64, in agreement with previous findings in dairy cows fed a basal diet supplemented with acetic or propionic acids [48]. Although Blaxter et al. [38,49] reported a lower K_f_ with high molar proportions of acetic acid [47], this was attributed to a mathematical error and after correction [19,50], values varied from 0.44 to 0.50, thus similar to those from the IIT [19]. Of added interest was the fact that in some of this work the E:P ratio was intentionally kept low to restrict glucose precursor supply [28]. One key question remained: if we assume that no differences in K_f_ existed between VFA, what caused the lower K_f_ for acetic acid observed by Blaxter?

In Armstrong and Blaxter’s original trial on a basal hay diet [38], no measurements were made regarding time spent eating and ruminating. Considerable muscle activity occurs during prehension, mastication and swallowing of feed, which can generate a 50% increase in oxygen consumption [51]. It was therefore concluded that the differences in K_f_ between concentrate and roughage diets were related to the mechanical costs of prehension, ingestion, mastication and not due to a lower efficiency of acetic acid utilisation per se [17]. This conclusion raised another question: how does ruminant metabolism respond to excessively high rumen acetic acid concentrations?

### 2.3. Relationship between VFA Composition, N Excretion and Blood Metabolite Concentrations

**Figure 2:**
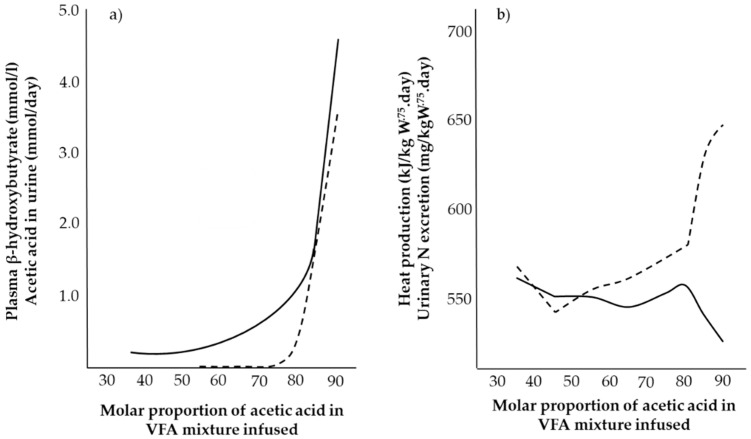
Effect of volatile fatty acid composition on (**a**) β-hydroxybutyrate (solid line) and acetic acid (dashed line) in urine, and (**b**) on heat production (solid line) and urinary N excretion (dashed line) of four steers. Adapted with permission from [17].

Changes in VFA composition are reflected in metabolic changes at animal level. Ørskov and MacLeod [17] measured blood plasma parameters, N excretion and heat production in growing steers infused to 1.5 maintenance energy requirement where the molar proportion of acetic acid ranged from 0.30 to 0.92 (varying inversely with propionic acid and butyric acid held constant at 0.08). The results are shown in Figure 2.

At high molar proportions of acetic acid (i.e., 0.75–0.90), plasma betahydroxibutyrate (BHB), urinary N and acetic acid excretion increase and heat production decreases (Figure 2). Due to the lack of glucogenic precursors, AA are partially oxidised during protein turnover leading to increased N excretion, in agreement with previous observations with sheep [38,52] and lambs [53,54]. The associated decrease in heat production [28,39] suggests that the excess acetic acid is not oxidised to yield heat but eliminated in the urine at no further energetic cost to the animal [17]. Additionally, plasma glucose and insulin decrease while non-esterified fatty acids (NEFA) increase [28,39]. Taken together, these changes may reflect the animal’s ability to acclimatise to its environmental conditions to efficiently manage its own energy reserves. To establish how the ruminant responds to fluctuating energy intake, experiments were then conducted to establish the metabolic response to deficiencies in energy and glucose supply.

### 2.4. Metabolic Changes Induced by Fasting and Key Role of Glucogenic Precursors

Glucose is a key nutrient required by the nervous system, foetus, mammary gland, muscle and for fat synthesis and turnover [7]. In ruminants, propionic acid is the main source of glucose as little glucose is absorbed directly from the diet [55]. Isotopic studies have shown that up to half [56] or more [57] of absorbed propionic acid is converted to glucose, reaching up to 90% in the fasted state [58].

On fasting, endogenous body reserves are mobilised leading to an increase in plasma ketones and NEFA, urinary N excretion and heat production [7,59,60]. Since these parameters revert to normal with glucose infusions [61], this suggests a glucose-deficient state in fasting. Fasting N excretion varies with age, growth and maturity with higher levels observed in young immature vs. mature fat steers (727 mg/kg W^.75^ vs. 539 mg/kg W^.75^ [60]) and young vs. adult castrate sheep [62]. In addition, large reductions of about 40% N excretion have been recorded with glucose infusions [63], illustrating the N-sparing effect of glucose on protein catabolism in ruminants [52]. However, a limit to this effect appears to exist since above a glucose infusion level of 5.5 g/kg W^.75^ no further N-sparing effect was observed [61]. Isoenergetic infusions of lipids also failed to elicit a N-sparing effect [63].

Urinary N excretion reflects the level of protein oxidation at tissue level with added glucose shown to spare relatively small amounts of protein catabolism in sheep and steers, even when large quantities of glucose (over 100 g) were infused [64]. Similar proportional reductions in fasting N excretion were observed by infusing casein-N in cattle [65] and sub-maintenance VFA in sheep [31,62] which accompanied reductions in heat production. Reductions in fasting heat production (per kg W^.75^) relate to the lower energetic costs of proteolysis, protein synthesis, lipolysis and ketogenesis [64,66]. Of the additional heat produced in fasting, 26% in cattle [60] and 22% in sheep [66] relates to the higher energy costs associated with protein turnover rather than lipid turnover [67].

### 2.5. Differences between K_f_ and K_m_

The efficiency of utilisation of metabolisable energy below maintenance (K_m_) is accepted to be higher than that above maintenance (K_f_) [7,17,68] and this is assumed in current NRC [69] and ARC [1] feeding systems. Maintenance energy requirements are based on fasting metabolism which is typically assessed after a period of diet restriction over four weeks followed by a four day fast [70,71], which reduces metabolic rates [72] and maintenance requirements by 10–50% [73]. This impacts the numerical value of fasting metabolism [7,17]. Furthermore, fasting metabolism varies within and between breeds of similar metabolic body weight [71] and fasting N excretion is up to 40% greater than basal VFA energy infusion [64]. For these reasons, the adequacy of applying fasting metabolism to conventional feeding regimes has been questioned [11,61]. Using data derived from a variety of diets, K_m_ values were quoted to range from 0.60 to 0.80 and K_f_ values from 0.25 to 0.50 [55].

**Figure 3:**
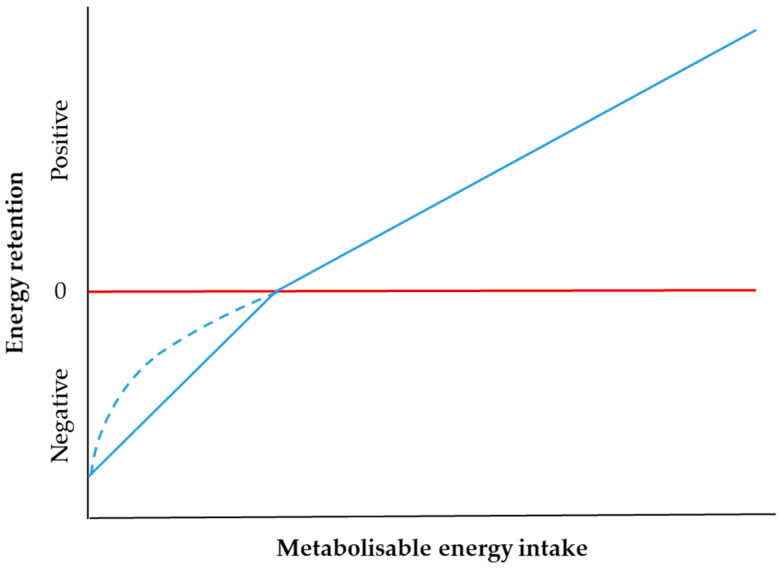
Effect of energy input on efficiency of utilisation above (K_f_) and below (K_m_) maintenance. The continuous blue line denotes the Agricultural Research Council assumption and the dashed blue line, the likely case. The red line denotes zero energy retention. Adapted with permission from [17].

To circumvent fasting, it was then suggested [7,17] to incorporate a baseline of approximately 0.3 of metabolisable energy maintenance requirements so that glucose precursor requirements could be met [7,11,17] (Figure 3). This proposal is not reflected in the current feeding models which assume a differential efficiency below and above maintenance, leading to the misleading assumption that feeds are used less efficiently above than below maintenance [11].

Ørskov and MacLeod [17] argued that the anomalies associated with fasting metabolism were an artefact of the method used to determine it, giving rise to differences in K_m_ and K_f_ (Figure 3) which they deemed as “dubious”. They also affirmed that K_f_ values between diets were closely related to the determined maintenance energy level, in agreement with recent affirmations by Cabezas-Garcia et al. [74] and that these values were prone to vary widely with even minor changes in heat production (of 10%).

### 2.6. Inter-Species and Physiology Stage Differences in Response to Glucogenic VFA or Glucose Infusions

Suggestions that glucose requirements are higher in cattle than sheep have been made [55,61]. These were based on the higher plasma BHB in fasted cattle than sheep and greater amount of glucose required to reduce plasma BHB to basal levels (5.5 g/kg W^.75^ in steers [61] vs. 1.6 g/kg W^.75^ in sheep [59]), higher glucose oxidation rate (41 kJ in steers vs. 35 kJ in sheep [75]), higher fasting N excretion in cattle (529 mg/kg W^.75^ vs. 466 mg/kg W^.75^ [61]) and greater response per unit of glucogenic VFA infusion (55:35:10) in cattle than in sheep [61]. Girdler et al. [76] using sheep maintained wholly by total intragastric infusion of VFA and casein-N to 1.1 times maintenance energy requirement, postulated a glucose requirement of 2.44 g/kg W^.75^ in sheep. Lobley [77] indicated a glucose requirement in fasted sheep of only 0.99–1.43 g/kg W^.75^.day but glucose utilisation rates appear to fall on fasting [78]. Lower glucose absorption rates have also been hypothesised in cattle vs. sheep [79] and wool growth is also known to have a high requirement for sulphur AA, some of which are glucogenic [80].

### 2.7. N Retention Responses

#### 2.7.1. Positive N Balance at Negative Energy Balance

**Figure 4:**
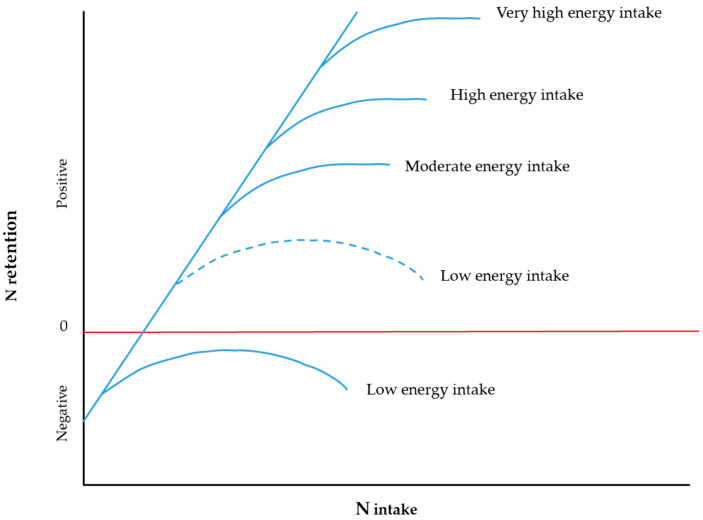
N retention response (linear blue slope) to varying energy:protein intake. The continuous lines represent the situation in the Balch model and the dashed curve “Low energy intake” denotes the more likely situation. The red line denotes zero nitrogen retention. Adapted with permission from [81].

Energy:protein ratios at whole animal level have been described by Balch [13] (Figure 4) to determine N requirements for ruminants and formulation of diets [1,68].

The slope of the linear portion of N retention to N intake curves depends on the similarity between the balance of AA required by the animal compared with the balance of the AA absorbed, reflecting catabolism of non-utilised AA. One of the key assumptions from this model is that at low energy intake protein retention is in all circumstances negative, irrespective of N intake [13]. This implies that AA are oxidised during protein turnover at times of undernutrition when AA requirements exceed dietary AA supply [82]. Comparable curvilinear responses in N retention to energy and protein intake have been reported with sheep [14] and other broadly similar linear-plateau models were proposed for growing lambs [83] and growing non-castrated male pigs [84].

Although the model of Balch [13] implies a negative N balance at very low energy input, evidence to the contrary was shown in dairy cows, sheep and steers (Table 5).

Energy balance was calculated for each energy infusion level as the sum of the VFA and casein-N energy equivalent (assuming maintenance energy requirements of 450 kJ/kg W^.75^) [19]. These findings, confirmed in lambs [23] and steers [65], proved that ruminants do not oxidise the supplied protein as a source of energy but instead utilise it to attain N balance and deposit tissue protein by oxidising their body fat to fuel the energy deficit, an effect also observed in humans [85]. Similar findings were also reported using the comparative slaughter technique with milk-fed lambs [86].

The N retention response curves to sub- and supra-maintenance VFA energy intakes were shown to vary between curvilinear [62,65] and linear [59,87], reflecting the diversity of animals used in the studies regarding stage of growth and maturity. Protein requirements of growing ruminants are determined by their capacity to deposit and retain body protein which in turn depends on factors such as genotype, sex and liveweight [84] and on a metabolic liveweight basis, are considerably higher in young lambs than young calves [2]. Curvilinear response curves suggest a variable efficiency of utilisation of absorbed AA unlike the linear response in the model of Balch [13] which assumes a fixed efficiency of utilisation. This linear response seems to contradict more recent studies which show that AA utilisation for milk protein synthesis varies depending on dietary AA supply [88,89] and AA composition [90,91,92].

#### 2.7.2. Influence of Animal Factors

**Figure 5:**
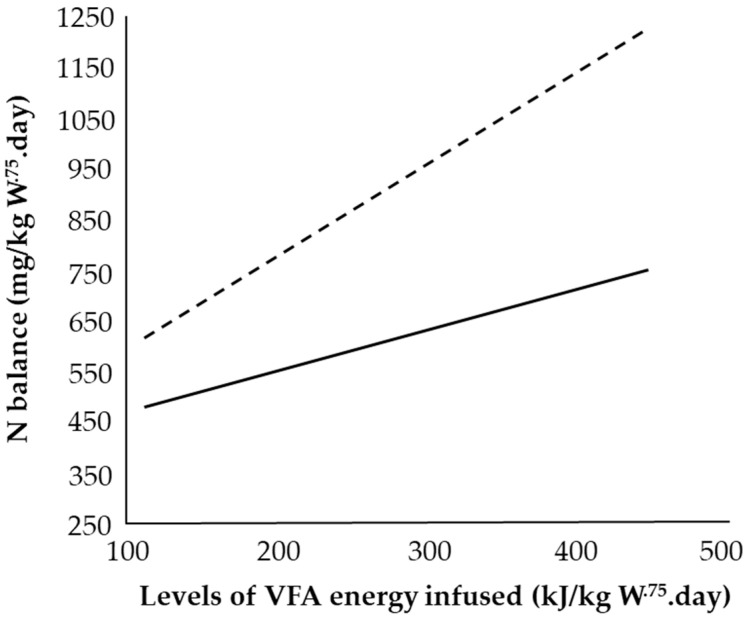
Mean N balance of heavy (solid line, *n* = 2) and light (dashed line, *n* = 3) sheep with incremental volatile fatty acids (VFA) infusion and a fixed infusion of 2250 mg casein-N/kg W^.75^.day. Heavy = 61 ± SD 2.2 kg liveweight. Light = 39 ± SD 4.5 kg liveweight. Adapted with permission from [59].

Animal-related factors such as prior feeding level and mature body weight influence N retention response. Using Suffolk-cross sheep given a sub-maintenance VFA infusion of a very high propionic acid mix (91 kJ/kg W^.75^) and increasing casein-N, Chowdhury et al. [59] reported a greater N balance in the lighter (39 ± SD 4.5 kg BW) vs. heavier (61 ± SD 2.2 kg BW) sheep (Figure 5). This was attributed to compensatory N repletion and an improved efficiency of N retention [23] of the light sheep due to a period of underfeeding before the trial [59]. Previous work by Hovell et al. [93] had shown a 52% increase in N retention (1103 vs. 724 mg N/kg W^.75^) after a period of protein depletion in sheep infused with VFA at 650 kJ/kg W^.75^ and casein-N at 2430 mg N/kg W^.75^. Additionally, despite being of similar age, the lighter sheep would presumably have had a lower body protein mass [94] and greater cell growth potential per unit mass [95]. As animals grow, whole-body protein synthesis increases but daily protein synthetic activity per unit tissue falls [95,96] and the contribution of both lean body mass and absolute protein turnover rates increase, which may mask the individual tissue/organ differences [95,97]. Thus, Chowdhury et al. [62] concluded that animals fed on a high plane of nutrition pre-trial used their accumulated endogenous fat reserves to substitute for exogenous VFA and exert a role on N retention.

#### 2.7.3. Influence of Glucogenic Precursors in VFA

**Figure 6:**
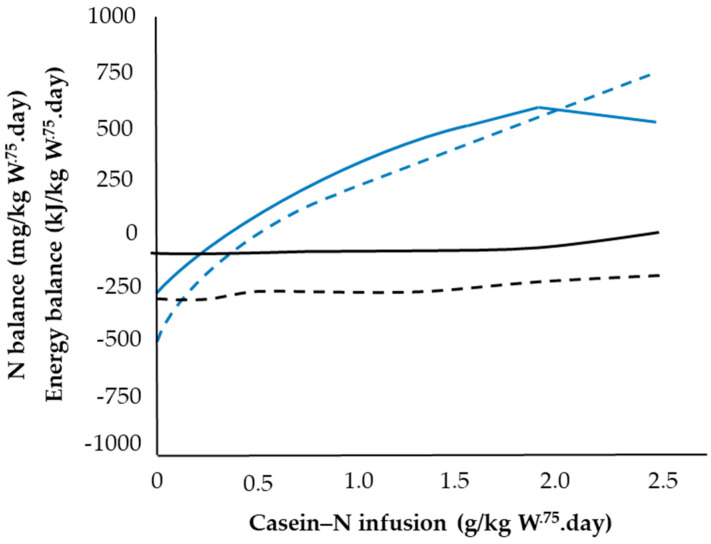
N (blue lines) and energy (black lines) balances of lambs given incremental casein with (solid lines) or without (dashed lines) 250 kJ/kg W^.75^.day from a volatile fatty acid mixture. Adapted with permission from [62].

Responses in N retention to exogenous VFA infusion could be related to both glucogenic and energy components of the VFA mixtures [59,61,62]. Chowdhury et al. [62] concluded a positive glucogenic effect on N retention using fasted lambs infused with sub-maintenance VFA (250 kJ/kg W^.75^) at low levels of casein-N (0−500 mg N/kg W^.75^; Figure 6).

**Figure 7:**
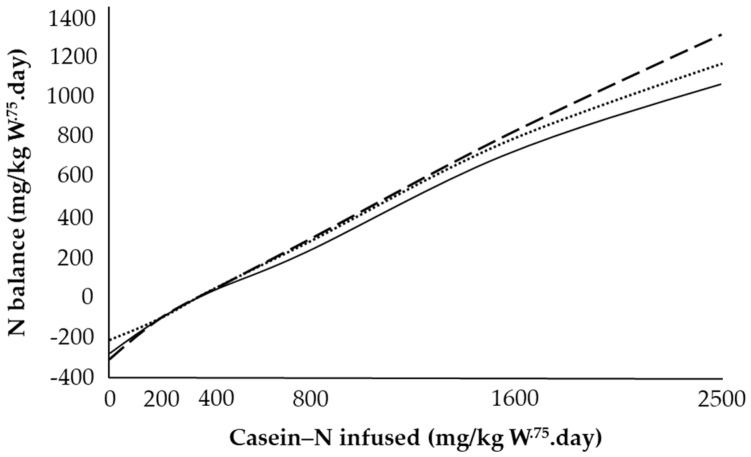
N balance response in relation to volatile fatty acid level and composition, at increasing casein-N infusion. Maintenance (450 kJ/kg W^.75^.day) on a low propionic acid mixture (solid line); maintenance on a high-propionic acid mixture (dashed line); 1.5 X maintenance on a low propionic acid mixture (dotted line). Adapted with permission from [61].

It was suggested that the N balance response reflected a glucose precursor supply (provided by the casein) rather than a specific exogenous energy effect per se since adequate endogenous energy reserves were available. Evidence to support this hypothesis was also shown in steers [61]. These authors, using two energy levels and two VFA mixtures high and low in glucogenic precursor supply infused to growing steers, observed a significantly higher coefficient of N retention for the high propionic acid mixture than the low propionic acid mixture at both energy levels (Figure 7). This indicated a likely role of glucogenic precursors on N retention response at the highest level of N infusion.

Studies using multi-catheterisation of the hepatic portal vasculature to measure nutrient fluxes post-absorption have also postulated a N-sparing effect of glucogenic precursors on amino acid gluconeogenesis at the level of the whole body [98], liver [99], splanchnic tissues [100], gastrointestinal tract [101] and mammary gland [88].

### 2.8. Endogenous Energy Reserves as a Fuel for Protein Deposition

#### 2.8.1. Growing Ruminants

At sub-maintenance energy intake, substantial mobilisation of body fat reserves is required to fuel protein anabolism and attain N balance [65]. Chowdhury et al. [59] using sub-maintenance energy infusions and infused casein-N at three times maintenance N requirements calculated body fat losses ranging from 1.29 to 4.03 kg in sheep and that for each kJ protein accretion 0.781 kJ of endogenous energy was used with an efficiency of 0.56. Previously, these authors reported a daily reduction of 100 g adipose tissue to equate to a N retention in lambs of 750 mg N/kg W^.75^.day at negative energy balance (approximately −180 kJ/kgW^.75^.day) [62]. These findings imply that body and carcass composition can be manipulated under this dietary regime and contradict other observations suggesting that body fat and protein depositions are positively correlated [15]. High levels of lean tissue gain can occur at these N infusion levels. Chowdhury et al. [65] infused fasted 400 kg steers with protein as the only exogenous nutrient and concluded that the N retention of 283 mg N/kg W^.75^.day at the highest infusion level (2000 mg N/kg W^.75^.day) equated to a weight gain of 0.8 kg/day, while only achieving an energy intake equivalent of 264 kJ/kg W^.75^.day (approximately half-maintenance energy requirements). Comparable levels of lean tissue gain (g/kg W^.75^.day) were reported in sheep [66]. Fattet et al. [102] also confirmed these findings using conventional diets over a 92-day N supplementation period. These authors showed that in lambs fed a basal NaOH-treated straw diet to sub-maintenance energy intake with or without 75 g/day of supplemental fishmeal, wool-free body protein was increased by 0.5 kg and body fat decreased by 1.5 kg [102]. These results are consistent with reduced hepatic protein oxidation while protein synthesis remains constant in lambs fed sub-maintenance protein [103]. However, a similar response was not observed when energy supply was reduced relative to protein supply [99]. Protein anabolism and catabolism occur continuously at sub-maintenance energy intakes, yet a 2% change in synthesis rate can alter protein accretion by 20–40% [97,104]. It has been suggested that the secretion of insulin-like growth factor I and growth hormone when exogenous amino acids are supplied to a ruminant in negative energy balance are responsible for these simultaneous processes of proteolysis and lipolysis [82] and insulin appears to reduce protein catabolism [105].

#### 2.8.2. Lactating Dairy Cows

High yielding dairy cows in early lactation and negative energy balance are particularly sensitive to increased AA supply in the small intestine [12,106], an effect also seen in dairy goats [107,108]. Incremental casein infusions were shown to double the negative energy balance of cows fed a mixed diet (from −20.5 to −41 MJ/day with 0 to 750 g/day casein-N), with a daily fat-corrected milk yield increase of 3 kg and milk protein concentration increase of 13% [109,110]. Relative to the amount of N infused, equal quantities were retained as milk protein and body protein, the latter to replenish the high levels of tissue labile body protein reserves mobilised in early lactation [111]. It was hypothesised that the high levels of body fat mobilised to support this yield increase was induced by AA supplied from the casein [110] rather than a glucogenic effect per se [12] or mediated by hormonal influence [112]. Indeed, lysine and methionine have been shown to be the most limiting AA for milk protein synthesis [80,113].

Casein infusion in early lactation appears to strengthen the homeorhetic drive [114] from mobilising body reserves towards milk secretion [115], a situation which seems to be reversed later in lactation. Homeorhetic regulation is maintained under hormonal control through alterations in circulating levels of hormones including insulin, growth hormone, prolactin, somatotropin and corticoids [114]. Large milk yield responses to casein infusions were also shown in mid lactation cows which are typically in positive energy balance [116] but where the output of milk energy far exceeds that supplied by the casein itself [112]. This additional energy may have derived from an increased efficiency of utilisation of energy for lactation (k_l_) or nutrient reallocation between body tissues and milk synthesis [116]. Responses to protein supply in late lactation cows were lower compared to those of mid lactation [117].

The adverse effects of energy and N deficits in high yielding cows on welfare and fertility have been documented [111,113,118]. Studies involving the feeding of glucogenic or lipogenic diets to early lactation cows have reported a reduced negative energy balance [119,120]. Conversely, attempts to reduce the protein deficit in early lactation by increasing the protein infusion level appear to further exacerbate the negative protein deficit [121]. Inadequate homeorhetic/homeostatic control in early lactation has been associated with metabolic ketosis [122,123].

## 3. Current Feeding Systems and Their Limitations

Although the IIT is no longer used due to animal welfare concerns, its key findings remain relevant today. However, these are not embodied in current feeding systems such as the ARC [1], NRC [69], INRA [124] or AFRC [68]. Firstly, fasting metabolism continues to be used to estimate the efficiency of utilisation of nutritionally balanced diets, although this appears conceptually incorrect since glucose requirements are not met and N excretion is elevated. Secondly, the ruminant’s ability to maintain a positive N balance while in negative energy balance, which has also been shown by the comparative slaughter technique, is not recognised. Given a ruminant’s genetic capacity for protein accretion, lean-body mass can be raised and carcase composition manipulated provided a rumen undegradable protein is fed, even when at sub-maintenance energy intake. Therefore, on extensive production systems, ruminant body fat reserves can be used as an “endogenous” energy source during the dry season when feed supply is sparse or of poor nutritive value. This strategy could be of particular importance to improve feed resource utilisation in developing countries and reduce the environmental impact of ruminant production systems. On intensive production systems, the findings question the need for feeding high energy diets at the finishing period (pre-slaughter) in lambs or steers and call for the current system of energy allowances to be reconsidered.

Although all major energy systems today continue to assume a higher K_m_ than K_f_*,* different equations are used to estimate efficiency of utilisation of metabolisable energy (maintenance, growth and lactation). Across systems, equations to predict maintenance energy requirements differ in energy units (net energy or metabolisable energy), method used (calorimetry or comparative slaughter) and apply to the predominant breeds in each geographical area (e.g., British and Continental breeds for AFRC [68] and *Bos taurus* and *Bos indicus* for NRC [125] in the USA and Canada). For AFRC [68] maintenance energy requirements continue to be based on fasting metabolism plus an allowance for physical activity and a different metabolic weight basis is used compared to INRA [124] (^.67^ vs. ^.75^). It is of interest that with respect to energy requirement predictions, the French net energy system distinguishes between the pre-ruminant and ruminant growth stages, different genotypes (dairy vs. beef origin) and, as with NRC [125], includes predictive equations for enteric methane production [74].

The curvilinear response between milk protein output and efficiency of metabolisable protein use [90,91] is not recognised in current feeding systems, which instead conform to the single limiting AA theory [91,92] by assuming a fixed 0.67 efficiency in metabolisable protein use for milk protein synthesis in NRC [69], 0.68 in the UK system [68] or 0.64 in both the Dutch [126] and French [124] systems. These efficiency figures also exceed the reported values of 0.5 [116], 0.65 [112], 0.5 [127] and 0.45 in finishing beef steers [128], against the ARC [1] value of 0.75. Regarding energy, current feed evaluation systems are incapable of predicting the differing responses to isoenergetic inputs of glucogenic or lipogenic diets to early lactation cows [91].

The ability of ruminants to use endogenous energy to fuel protein deposition [59,62] has led to calls for the current system of energy allowances to be reconsidered [11] since this concept questions the need for high energy diets for lambs or beef cattle in the finishing stage pre-slaughter. The potential to improve carcase protein:fat ratio by limiting energy intakes in the finishing period has also been shown in other studies [129,130].

Feeding rumen-protected protein to high yielding dairy cows in early lactation does not appear to alleviate the extent of the protein deficit but glucogenic diets may help reduce the energy deficit [119]. On a global scale, efficiency of production is not solely restricted to high genetic merit progeny fed high quality diets but can equally exist with indigenous breeds fed on local, low quality feed resources and where the choice of feed supplements will depend on local resources and presence (or absence) of industrial by-products.

Recent years have shown the replacement of a factorial with a more mechanistic model of rationing based on studies to predict the flux of energy yielding nutrients [131,132] and protein [132,133] post-absorption. These systems describe constant turnover functions such as protein turnover in the muscle and viscera and triglyceride turnover in adipose tissue while assuming that requirements are a function not only of the present state but also prior feeding regime [134]. The application of such models on farm is however constrained by their high errors in predicting performance (liveweight gain, milk yield/day [131,134]).

## 4. Conclusions

The IIT was invasive in nature but versatile in allowing E:P ratio in ruminants to be studied in absolute terms, without the confines imposed by a functioning rumen. However, infusions were controlled, no feed or rumen microbiota was propelled through the gut, only small animal numbers were used and at sub-maintenance rather than supra-maintenance infusion levels. The main VFA (acetic, propionic and butyric acids) are utilised with a similar efficiency of typically 60%. General curvilinear and linear responses exist in N retention to increased energy and protein intakes. Fasting metabolism is an inappropriate basis to measure the utilisation of nutritionally balanced diets due to a lack of glucogenic precursors and such estimates should be made at a basal level of intake (approximately one third maintenance energy requirements) to ensure glucose requirements are met. Ruminants have the innate ability to distinguish between protein and energy yielding nutrients and prioritise essential nutrients to maintain tissue protein by using body fat reserves to “fuel” the energetic costs of protein deposition. The classical concept of E:P ratio does not consider the potential role of endogenous energy reserves. The complexity of energy:protein metabolism in the ruminant implies that predicting nutrient requirements of ruminants is difficult and no one technique including the IIT can accurately quantify the true response to nutrients fed. This difficulty is also reflected in the large errors associated with predicting performance in modern mechanistic nutrient modelling systems.

## Figures and Tables

**Table 1:** Maintenance metabolisable energy requirement (kJ/kg W^.75^) estimates by IIT.

Ruminant Species	Method	Metabolisable Energy	Reference
Lambs	Intragastric infusion	450	[19]
Lambs	Comparative slaughter	420	[28,29]
Sheep and steers	Calorimetry	420–460	[1]

**Table 2:** Maintenance N requirement (mg/kg W^.75^) determined by IIT.

Ruminant Species	Urinary N	Faecal N	Total N	Reference
Lambs	427	ND	427	[30]
Lambs	340	ND	340	[33]
Steers	403	ND	406	[31]
Dry cows	295	25	325	[31]
Pregnant cows (233 d)	283	ND	283	[31]

ND: Not determined.

**Table 3:** Efficiency of utilisation of volatile fatty acids (VFA) for energy retention (K_f_) in ruminants.

Ruminant Species (Number)	VFA Composition (C_2_:C_3_:C_4_) ^1^	VFA Infused (kJ/kg W^.75^)	K_f_ ^2^	Reference
Sheep (3)	Acetic acid ^3^	450 ^4^	0.67	[38]
	Propionic acid ^3^		0.44	
	Butyric acid ^3^		0.38	
Sheep (48)	45:45:10	450	0.64	[19]
	55:35:10	900 ^5^	0.57	
	65:25:10		0.61	
	75:15:10		0.61	
	85:50:10		0.59	
Steers (4)	46:46:08	675 ^6^	0.55	[39]
	56:36:08		0.55	
	66:26:08		0.55	
	76:16:08		0.55	
	81:11:08		0.55	
	86:06:08		0.55	
	91:01:08		0.55	
Steers (4)	36:56:08	450	0.61	[28]
	46:46:08	675	0.61	
	56:36:08	900	0.59	
	66:26:08		0.59	
	76:16:08		0.59	
	81:11:08		0.59	
	86:06:08		0.59	
	91:01:08		0.59	
Steers (3)	65:27:08	450	0.64	[40]
	49:43:08	675	0.64	

^1^ Acetic acid:propionic acid:butyric acid ratio. ^2^ Estimated value based on maintenance energy (450 kJ/kg W^.75^). ^3^ Infused as single individual VFA. ^4^ 1 X maintenance energy requirement. ^5^ 2 X maintenance energy requirement. ^6^ 1.5 X maintenance energy requirement.

**Table 4:** Efficiency of utilisation of energy for retention (K_f_) and lactation (K_l_) on forage-based diets from feeding trials.

Ruminant Species	Type of Diet	K_f_	K_l_	Reference
Sheep	Dried autumn grass	0.45		[42]
	Dried autumn grass + casein	0.57		
Sheep	Ryegrass + barley	0.36		[43]
	Ryegrass + maize	0.42		
	Clover + barley	0.44		
	Clover + maize	0.50		
Cattle	Setaria grass	0.17		[44]
	Pangola grass	0.28		
Lactating cows	Low grain intake		0.68	[45]
	High grain intake		0.77	
Brahman bulls	Fresh maize (restricted)	0.32		[41]
	Fresh napier (restricted)	0.25		
	Maize *ad libitum*	0.20		
	Napier *ad libitum*	0.15		
Dry cows	Autumn pasture	0.34		[46]
	Autumn pasture + maize silage	0.47		
	Autumn pasture + pasture silage	0.50		
	Autumn pasture + maize grain	0.38		
	Autumn pasture + palm kernel meal	0.61		
Cattle and sheep	Hay:maize (100:0)	0.29		[47]
	Hay:maize (80:20)	0.34		
	Hay:maize (60:40)	0.43		
	Hay:maize (40:60)	0.47		
	Hay:maize (20:80)	0.54		
	Hay:maize (0:100)	0.61		

**Table 5:** Level of gross energy infusion (kJ/kg W^.75^) at which zero N balance was attained.

Ruminant Species (Number)	Gross Energy Infused	Type of Infusion ^1^/Day	Reference
Lactating cows (2)	281 (SE ^2^ 26)	Gross (VFA + protein) energy of 675 kJ/kg W^.75^ Fixed casein N of 750 mg/kg W^.75^ Gross infusion reduced to energy value of casein N (118 kJ/kg W^.75^)	[5]
	115 (SE 16)	460 kJ/kg W^.75^ + 972 mg N/kg W^.75^ 450 kJ/kg W^.75^ + 0 mg N /kg W^.75^ 225 kJ/kg W^.75^ + 0 mg N/kg W^.75^ 0 kJ/kg W^.75^ + 0 mg N/kg W^.75^ 147 kJ/kg W^.75^ + 938 mg N/kg W^.75^	
Steers (4)	154 (SE 38)	450 kJ/kg W^.75^ + 1 g N/kg W^.75^ 450 kJ/kg W^.75^ + 0 g N/kg W^.75^ 0 kJ/kg W^.75^+ 0 g N/kg W^.75^ 150 kJ/kg W^.75^ + 1 g N/kg W^.75^ 250 kJ/kg W^.75^ + 1 g N/kg W^.75^ 350 kJ/kg W^.75^ + 1 g N/kg W^.75^ 450 kJ/kg W^.75^ + 1 g N/kg W^.75^	
Lambs (4)	162 (SE 29)	VFA energy of 0, 120, 230, 340, 450, 560 and 670 kJ/kg W^.75^ Fixed casein N of 972 mg N/kg W^.75^ Infusions stepped up or down from starting level of 340 kJ/kg W^.75^	[28]
	150 (SE 22)	VFA energy of 0, 75, 150, 250, 350, 450, 575 and 700 kJ/kg W^.75^ Fixed casein N of 530 mg N/kg W^75^ and 1060 mg N/kg W^.75^ Infusions stepped up or down from starting level of 450 or 575 kJ/kg W^.75^	
Lambs (8)	330 + 250 VFA energy 157 + 0 VFA energy	VFA energy of 0 or 250 kJ/kg W^.75^ Casein-N of 0, 250, 500, 750, 1350, 1950 and 2550 mg N/kg W^.75^ Casein-N infusions stepped up or down	[62]
Lambs (10)	195	Fixed VFA energy of 91 kJ/kg W^.75^ Incremental casein N of 0, 250, 500, 750, 1500, 2250 and 3000 mg N/kg W^.75^	[59]
	205	Gross (VFA + protein) energy of 91 kJ/kg W^.75^ Incremental casein N of 0, 250, 500, 750, 1500 and 2250 mg N/kg W^.75^	
Steers (2) fasting	264	Zero VFA energy Incremental casein N of 0, 250, 500, 750, 1000, 1500 and 2000 mg N/kg W^.75^	[65]

^1^ Molar proportions of 0.65, 0.25, 0.10 acetic, propionic and butyric acids in all cases. ^2^ Standard error.
